# The role of corepressor HOS15-mediated epigenetic regulation of flowering

**DOI:** 10.3389/fpls.2022.1101912

**Published:** 2023-01-10

**Authors:** Li-Jun Huang, Yukun Wang, Zeng Lin, Dong Jiang, Yong Luo, Ning Li

**Affiliations:** ^1^ State Key Laboratory of Cultivation and Protection for Non-Wood Forest Trees, College of Forestry, Central South University of Forestry and Technology, Changsha, China; ^2^ School of Chemistry and Environmental Science, Xiangnan University, Chenzhou, China; ^3^ Key Laboratory of Forest Bio-resources and Integrated Pest Management for Higher Education in Hunan Province, Central South University of Forestry and Technology, Changsha, China

**Keywords:** epigenetics, transcriptional corepressor, HOS15, GIGANTEA, flowering

## Abstract

Regulation of gene expression underpins gene function and is essential for regulation of physiological roles. Epigenetic modifications regulate gene transcription by physically facilitating relaxation or condensation of target loci in chromatin. Transcriptional corepressors are involved in chromatin remodeling and regulate gene expression by establishing repressive complexes. Genetic and biochemical studies reveal that a member of the Groucho/Thymidine uptake 1 (Gro/Tup1) corepressor family, HIGH EXPRESSION OF OSMOTICALLY RESPONSIVE GENE 15 (HOS15), is recruited *via* the evening complex (EC) to the *GIGANTEA* (*GI*) promoter to repress gene expression, and modulating flowering time. Therefore, HOS15 connects photoperiodic pathway and epigenetic mechanism to control flowering time in plants. In addition, growing body of evidence support a diverse roles of the epigenetic regulator HOS15 in fine-tuning plant development and growth by integrating intrinsic genetic components and various environmental signals.

## Introduction

Plants are the most fundamental organisms on earth that sustain global ecosystem and food production (Raven, 2019; Eckardt et al., 2022). The changing environmental conditions, however, has threatened the plants. Unfortunately, land plants as sessile organisms are unable to choose or change environment for their development and growth. Plants as multicellular organisms are strikingly plastic and have evolved the ability to coordinate their growing phenotype in response to the changing environment (Pierik et al., 2021). For instance, the timing of flowering is a highly plastic phenomenon. A variety of external signals, such as day length and temperature, may induce endogenous physiological changes and accelerate flowering, the critical switch from vegetative to reproductive growth (Srikanth and Schmid, 2011; Freytes et al., 2021). Cells of a complex multicellular organism are homogeneous at genomic level but are physiologically and functionally heterogeneous due to the dynamic expression of genes. The spatiotemporal regulation of gene expression, which determines the timing and pattern of gene function, supports the phenotypic plasticity of plant development and growth (Nicotra et al., 2010; Jong and Leyser, 2012). Epigenetic regulation of chromatin status has become one of the most exciting frontiers of gene expression research in plant science. Epigenetic modification influences chromatin conformation and related transcriptional states by means of DNA methylation and histone modifications (Jaenisch and Bird, 2003; Gibney and Nolan, 2010; Eriksson et al., 2020).

Corepressors are transcriptional regulators (Courey and Jia, 2001). These transcriptional corepressors epigenetically repress target genes by forming a multi-protein complex with other transcription factors, adapters and accessory proteins. HIGH EXPRESSION OF OSMOTICALLY RESPONSIVE GENE 15 (HOS15) belongs to the most prominent and evolutionarily conserved Groucho/Thymidine uptake 1 (Gro/Tup1) family corepressors, which include the Gro protein in nematodes, the Tup1 protein in yeasts, and the transducin beta-like protein 1 (TBL1) protein in mammals. They contain a N-terminal glutamine (Q)-rich motif and a C-terminal region comprising multiple-repeat of approximately forty amino acids enriched for tryptophan-aspartic acid dipeptide (WD40). In Arabidopsis, at least 13 members of the Gro/Tup1 family corepressor have been identified, including TOPLESS (TPL) and four TPL-related proteins (TPRs), LEUNIG (LUG) and LUG HOMOLOG (LUH), HOS15 and other uncharacterized members (Liu and Karmarkar, 2008; Lee and Golz, 2012). Among them, the TPL/TPR sub-family proteins selectively interact with HDA19. The TPL-HDA19 module has been implicated in regulating a broad range of developmental and environmental processes by association with different transcription factors (Long et al., 2006; Causier et al., 2012; Krogan et al., 2012; Wang et al., 2013; Oh et al., 2014; Plant et al., 2021). Thus, it was not surprising when HOS15 was found to be involved in a variety of biological processes, like TPL/TPRs, through connecting with histone deacetylases. However, neither HOS15 nor TPL/TPRs contains a DNA binding domain, therefore, the corepressors require additional patterners to reach promoter regions of target genes. Recent study revealed that HOS15 was recruited by a protein complex consisting of transcription factors to the promoter region of *GIGANTEA* (*GI*) to repress photoperiodic pathway which accelerates Arabidopsis flowering under long days (Hammond, 2019; Park et al., 2019). This review focus on the molecular mechanism of HOS15-mediated epigenetic regulation of flowering and highlights the role of HOS15 in integration of both endogenous physiological cues and exogenous environmental stimuli stresses.

## HOS15 is involved in regulating photoperiodic flowering

Previously, an elegant high-throughput genetic screening system was designed to decipher the complex osmotic and cold stress signaling cascade in Arabidopsis using the chimeric gene construct as reliable reporter, in which the stress-responsive *RD29A* (also known as *COR78* for *cold regulated 78*) gene promoter was used to control the expression of the firefly luciferase gene that quantitatively and faithfully reflecting the *RD29A* promoter activity (Ishitani et al., 1997). A large number of different categorized mutants with altered expression of the reporter gene was identified, such as *cos*, *los*, and *hos*, named after constitutive, low, and high expression of osmotically responsive genes, respectively. Interestingly, some of those mutants also displayed a plethora of developmental phenotypes, for examples, the *hos1* mutant with defect in a RING finger E3 ubiquitin ligase coding gene (gene locus: AT2G39810) flowers remarkably earlier than wild-type (Jung et al., 2012; Lee et al., 2012); while the *hos2* [also designated *fiery1* (*fry1*) for strong expression of firefly luciferase reporter activity] mutant with defect in a bifunctional phosphoadenosine phosphatase coding gene (AT5G63980) has fewer lateral roots, shorter petioles, crinkly leaves, and flowers later (Xiong et al., 2001; Chen and Xiong, 2010). The pleiotropic developmental phenotypes of the *HOS* family mutants manifest that the developmental and stress response signaling pathways are genetically interactive.

**Figure 1:**
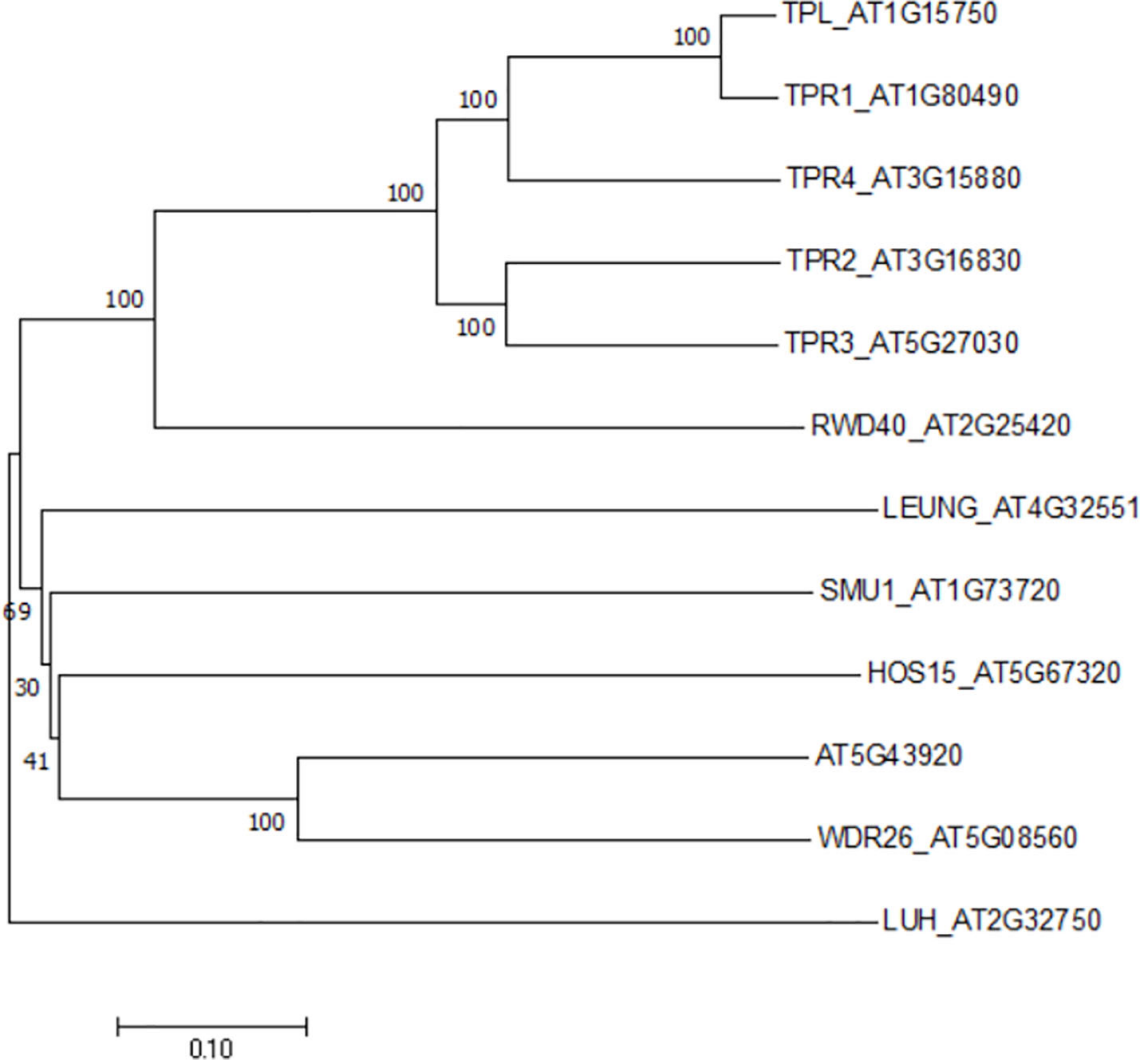
A phylogenetic tree of the Gro/Tup1 corepressor gene family in Arabidopsis. The gene names were followed by TAIR locus numbers. An unrooted neighbor- joining tree was generated by MEGA X with full-length sequences (1, 000 bootstrap replicates).

The casual gene responsive for *hos15* mutant phenotype was identified to encode a WD40-repeat protein belonging to the Gro/Tup1 family corepressor (Zhu et al., 2008), which includes a dozen of members in Arabidopsis (**Figure 1**). However, neither HOS15 interacting proteins nor its role in plant development has been described. While studying the molecular function of HOS15, Park et al. noticed, by coincidence, that *HOS15* loss-of-function (*hos15*) plants flower earlier than wild-type (WT) plants. By contrast, the flowering time of *HOS15* gain-of-function plants become delayed. Hooked by this robust and interesting phenotype, the authors set out to elucidate the molecular mechanism underlying *HOS15*-mediated control of flowering time.

Regulation of flowering time has been a hot topic studied intensively over the past 100 years. These studies implicated that the initiation of flowering was controlled by multiple genetically defined pathways that integrate both exogenous environmental cues and endogenous developmental signals (Andrés and Coupland, 2012). The vernalization pathway refers to the establishment of competence to flower after a prolonged exposure of plants to cold. The gibberellic pathway refers to the requirement of the phytohormone, gibberellin (GA), to promote normal flowering patterns. The photoperiodic pathway refers to the regulation of flowering by day length and light quality (Shim et al., 2017). *CONSTANS* (*CO*) plays a central role in the photoperiodic flowering pathway, in which the rhythmic expression pattern of *CO* is regulated by *GIGANTEA* (*GI*) (Fowler et al., 1999; Park et al., 1999; Mizoguchi et al., 2005; Park et al., 2013). The endogenous regulators that promote flowering in a photoperiod- and GA-independent way often referred to as the autonomous pathway. To first crack the mystery by determining which pathway was affected in *hos15* mutants, a high-throughput RNA sequencing experiment was performed to identify if any genes of specific pathway were differentially expressed in *hos15* mutants when compared to WT. The expression of key floral pathway integrator genes, such as *FLOWERING LOCUS T* (*FT*) (Corbesier et al., 2007), *SUPPRESSOR OF OVEREXPRESSION OF CONSTANS 1* (*SOC1*) (Samach et al., 2000; Yoo et al., 2005), *APETALA1* (*AP1*) (Mandel et al., 1992), *AGAMOUS-LIKE 19* (*AGL19*) and *AGL24* (Yu et al., 2002; Schönrock et al., 2006), was higher in *hos15* mutants. This unbiased analysis also revealed that the expression of photoperiodic flowering pathway genes, including *GI*, *CO*, and *FLAVIN-BINDING KELCH REPEAT F-BOX 1* (*FKF1*) (Ito et al., 2012), was specifically upregulated; whereas the expression of other flowering pathway genes was unchanged in *hos15* mutants. The results strongly indicate that the photoperiodic pathway is provoked in *hos15* mutants leading to early flowering.

## HOS15 is recruited to the *GI* promoter by the evening complex

As mentioned above, genes such as GI and CO are involved in promoting flowering in response to photoperiod. GI is a key circadian integrator and a master regulator of CO expression. Under long-day condition, light promotes the expression of GI and a ubiquitin-ligase FKF1 peak at the same time, leading to the optimal formation of the GI–FKF1 complex, which, in turn, promotes the degradation of transcriptional repressors of CO (Imaizumi et al., 2005; Sawa et al., 2007). CO is a floral activator that directly activates FT expression. Therefore, GI controls flowering time through regulating the CO-FT module. Indeed, GI protein stability is controlled by light and circadian clock. The clock-associated protein EARLY FLOWERING 3 (ELF3) acts as a substrate adaptor, enabling a RING-type E3 ubiquitin-ligase CONSTITUTIVE PHOTOMORPHOGENIC 1 (COP1) to target GI for the ubiquitin-26S proteasome-mediated proteolysis (Yu et al., 2008). Interestingly, *GI* transcript levels were consistently increased in *elf3* mutants, which displays an early-flowering phenotype (Kim et al., 2005). Therefore, ELF3 controls GI protein abundancy at both transcriptional and post-transcriptional levels. But how ELF3 represses GI expression remain mysterious. ELF3 is a constituent component of a protein complex (called the evening complex [EC]), consisting of ELF3, ELF4 and a transcription factor, LUX ARRHYTHMO (LUX, also termed PHYTOCLOCK1), which directly binds to the *GI* promoter through LUX binding site (LBS) to repress *GI* transcription in the night (Mizuno et al., 2014).

Since *GI* transcription is repressed by both the evening complex and the transcriptional corepressor HOS15, the authors wondered whether the evening complex brings HOS15 to the *GI* promoter. A pilot chromatin Immunoprecipitation (ChIP) assay revealed that like LUX, HOS15 associated with the *GI* promoter *via* binding to the same LBS regions (Park et al., 2019). This result promoted the author to further investigate whether HOS15 can bind to the evening complex. Both *in vitro* Y2H and *in vivo* experiments confirmed that HOS15 interacted with the evening complex components, LUX and ELF3 as well. A gel-filtration analysis showed the presence of a high-order complex containing HOS15-LUX-ELF3 (Park et al., 2019). Further ChIP assays with loss-of-function mutants indicated that HOS15 and LUX were interdependent in terms of binding to *GI* promoter; HOS15 was unable to bind to *GI* promoter in *lux* mutants, and *vice versa*. Thus, it become clear that the transcriptional corepressor HOS15 was recruited to the *GI* promoter *via* direct association with the evening complex to silence GI expression. Now, there comes another question where is the real core repressor of the repression-complex established on the target promoter.

## HOS15 interacts with a histone deacetylase HDA9

HOS15 contains a WD40 repeat motif and is ortholog to TBL1 in mammals (Zhu et al., 2008). TBL1 coordinates the formation of transcriptional repression complexes comprising either nuclear receptor corepressor (N-CoR, also known as NCOR1) or silencing mediator for retinoic acid and thyroid receptor (SMRT, also known as NCOR2), which function in chromatin modification of target gene promoter regions. TBL1 selectively deploys histone deacetylases (HDACs) of the Reduced potassium dependency 3 (Rpd3)-like superfamily that is broadly conserved in all eukaryotes (Yoon et al., 2005). HDACs are responsible for catalyzing histone deacetylation modification. In Arabidopsis genome the class I Rpd3-like HDAC family is composed of HDA6, HDA7, HDA9, and HDA19. Interestingly, HDA9 and HDA19 were found in a mass spectrometry-based proteomic approach aimed to identify HOS15-interacting proteins (Park et al., 2018a).

**Figure 2:**
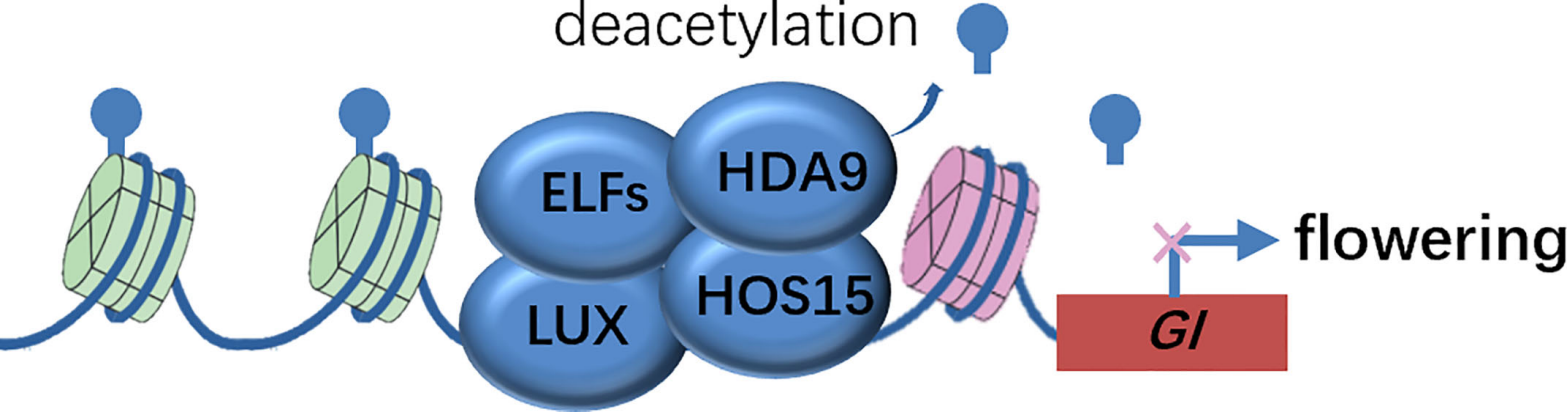
Molecular model of HOS15-mediated higher-order repressive complex in *GI* promoter region to inhibit *GI* expression via histone deacetylation. Inhibition of *GI* expression delays flowering time. EFLS, EARLY FLOWERINGS; GI, GIGANTEA; HDA9, HISTONE DEACETYLASE 9; HOS15, HIGH EXPRESSION OF OSMOTICALLY RESPONSIVE GENE 15; LUX, LUX ARRHYTHMO.

Genetic evidence that both *hda9* single and *hda9 hos15* double mutants phenocopied the early-flower phenotype of *hos15* mutants, suggested that HDA9 was a strong candidate that functioned in association with HOS15. The authors biochemically confirmed that HDA9 interacted with HOS15 and HDA9 bond to *GI* promoter regions in a HOS15-dependent manner, by harnessing co-IP and ChIP analysis, respectively. Consistently, the Histone H3 on the *GI* locus was hyper-acetylated in *hos15* mutants. Therefore, it appears evident that the EC complex recruits the HOS15-HDA9 module to the *GI* promoter and represses *GI* transcription by modulating the epigenetic status, and thereby delaying flowering time (**Figure 2**). The corepressor HOS15 connects photoperiodic pathway regulated by GI and epigenetic modification at histones catalyzed by HDA9 to control flowering time in plants.

As broached above, the Arabidopsis Rpd3-like HDACs in general were believed to be involved in different plant biological processes by regulating gene expression through histone deacetylation. The mechanisms by which HDACs are selectively recruited to specific target gene loci in chromosome are not well understood. Notably, HDACs may have non-histone substrates (Zheng et al., 2020; Xu et al., 2021). In an immunoaffinity purification assay to identify protein complex associated with HDA9 revealed that the WRKY53 transcription factor could co-purified with HDA9 (Chen et al., 2016). A domain-swapping experiment further demonstrated that the deacetylase domain of HDA9 mediated the interaction with WRKY53. Biochemical analysis using an anti-acetylated lysine antibody revealed that WRKY53 lysine acetylation levels were increased and decrease in HDA9 loss- and gain-of-function mutants, respectively, as compared to the wild-type background (Chen et al., 2016). HDA9 might inhibit WRKY53 transcriptional activity through deacetylation modification, thereby regulating downstream gene expression. Interestingly, through a large-scale protein acetylome analysis, ribosomal proteins were identified as substrates of HDA714, a member of Rpd3-like HDACs in rice (Xu et al., 2021). Contrary to the long-held belief that histone deacetylases act only on the tails of histone proteins, HDACs might form distinct protein complexes and regulate gene expression through various mechanisms in different physiological contexts, which offers the possibility of complicate crosstalk and delicate balance among diverse signaling pathways.

## The loose ends: A versatile role of HOS15 in development and more

The role of HOS15-mediated regulation of flowering time has been clearly elucidated in Arabidopsis. However, as a universal corepressor, it is not unexpected that HOS15 is involved in a myriad of physiological processes through interacting with corresponding transcription factors.

Mutation of *HOS15* massively affects histone acetylation and methylation levels, which subsequently alters expression of a large number of genes involved in developmental programs and environmental responses (Mayer et al., 2019). In addition to regulate flowering time, Suzuki et al. reported that HOS15 plays a critical role in the specification and definition of aerial lateral organ size by promoting cell proliferation during leaf developmental program (Fujikura et al., 2009; Suzuki et al., 2018). HOS15 also promotes age-associated leaf senescence *via* differential regulation of senescence- and photosynthesis-related genes (Zareen et al., 2022).

HOS15 not only regulates plant development and growth but also participates in plant response and adaptation to environmental changes. Several lines of evidence supported that HOS15 interacts with histone deacetylase 2C (HD2C) and induces HD2C degradation *via* the CULLIN4-based E3 ubiquitin ligase in a cold-dependent manner, which releases the expression of cold responsive genes to enhance cold tolerance (Zhu et al., 2008; Park et al., 2018b; Lim et al., 2020; Lim et al., 2021). HOS15 negatively regulates Snf1-Related Kinases (SnRKs) activity of the abscisic acid (ABA) signaling pathway (Ali and Yun, 2020; Khan et al., 2022). In addition to be involved in abiotic stress response, HOS15 also regulates plants resistance to pathogen infections. Shen et al. revealed that HOS15 sophisticatedly manipulates plant defense response by destabilizing the transcriptional activity of NONEXPRESSOR OF PATHOGENESIS-RELATED GENES 1 (NPR1), the master regulator of plant immunity networks (Shen et al., 2020). Yang et al. revealed that HOS15 intercepts plant defense response by repressing the expression of nucleotide-binding leucine rich repeat/NOD-like receptors, which recognize invading pathogen effectors to provoke effector-triggered immunity; therefore, mutation of *HOS15* confers plants more resistant to pathogen infection (Yang et al., 2020). They also noticed that mutation of HOS15 results in enlarged siliques, the seed capsules of cruciferous plants (Mayer et al., 2019; Shen et al., 2020). In addition to acetylation, histone methylation, another type of post-translational modification may also be involved in fine-tuning the trade-off between growth and defense in plants. For example, the members of the histone methyltransferase family, SET DOMAIN GROUP 8 (SDG8) and ARABIDOPSIS TRITHORAX 1 (ATX1, also known as SDG27), positively regulate plant immune response to phytopathogens while actively repress plant flowering time (Berr et al., 2010; S., Y., Kim et al., 2005; Zhao et al., 2005; Pien et al., 2008; Lee et al., 2016). Regulation of histone modification is a gigantic platform for different pathways convergence and interaction that deserves further exploration.

**Figure 3:**
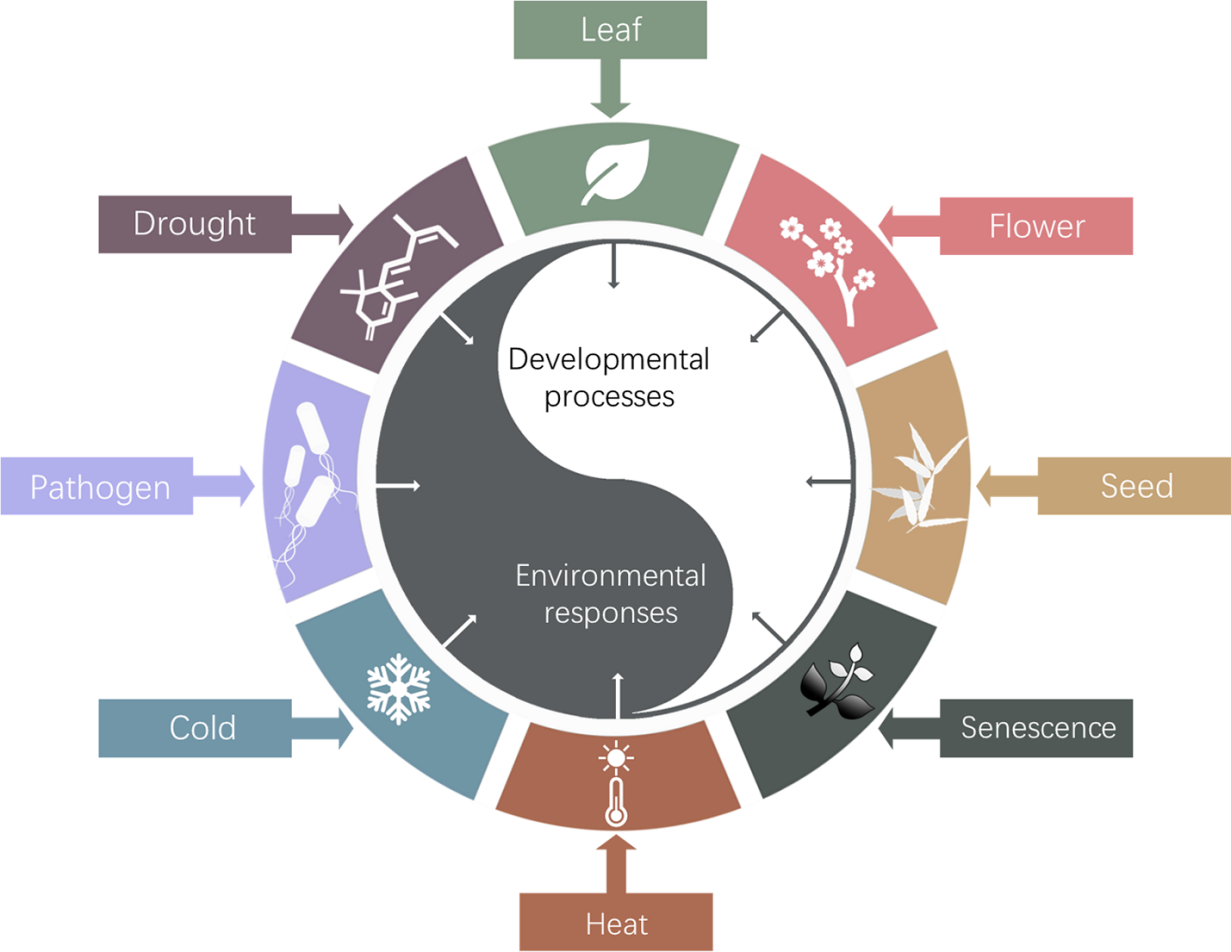
Schematic diagram of HOS15 regulated developmental processes and response to environmental stresses. So far, HOS15 was found to play multiple roles in plant organ development, such as leaf, flower, seed and senescence; and plant response to various abiotic and biotic stresses, such as drought, temperature, and pathogens.

The so far identified biological roles of Arabidopsis HOS15 were summarized in **Figure 3**. Studies of HOS15 function will elucidate the molecular mechanism underlying how plants epigenetically regulate development and environmental adaptations and trade-off between these two processes, which will eventually provide insights on how to develop novel high quality, high yield and stress resilient crops by innovative bottom-up molecular breeding technologies to sustain agriculture under global climate change.

## Author contributions

L-JH, YW, and NL: conceptualization. L-JH, DJ, and NL: literature review. L-JH, ZL, and NL: writing—original preparation. NL and YL: writing—review and editing. L-JH, YW, DJ, and NL: design and revision of the images. All authors have read and agreed to the final version of the manuscript.
